# The Community of Practice: A Method for Cooperative Learning of Occupational Health and Safety Inspectors

**DOI:** 10.3390/ejihpe11040091

**Published:** 2021-10-12

**Authors:** Luisella Gilardi, Maurizio Marino, Lidia Fubini, Antonella Bena, Elisa Ferro, Silvano Santoro, Eleonora Tosco, Osvaldo Pasqualini

**Affiliations:** 1 Documentation Centre for Health Promotion, Local Health Authority TO3, Grugliasco, 10095 Torino, Italy; lidia.fubini@dors.it (L.F.); antonella.bena@dors.it (A.B.); elisa.ferro@dors.it (E.F.); silvano.santoro@dors.it (S.S.); eleonora.tosco@dors.it (E.T.); 2 Epidemiology Department, Local Health Authority TO3, Grugliasco, 10095 Torino, Italy; maumarino1954@gmail.com (M.M.); osvaldo.pasqualini@epi.piemonte.it (O.P.)

**Keywords:** occupational safety and health, prevention, training methods, evaluation

## Abstract

Background: Workplace injuries in Italy still occur despite laws and safety norms. We need to understand the causes rooted in the context and social conditions, and need to improve the practice of Occupational Safety and Health (OSH) inspectors of the Workplace Safety and Prevention Services (WSPS) of the Italian regional health boards. The aims of this study were to describe the setting up of a Community of Practice (CoP) for the production of best practices for injury prevention and to evaluate the motivation of OSH inspectors for participating in the CoP and the effects of CoP participation on their professional practice. Methods: Two workplace injury stories underwent peer review during each CoP meeting. We evaluated the CoP using a focus group and a questionnaire. Result: Between 2014 and 2021, the CoP met in 18 workshops. Over the 8-year period, the CoP grew from 20 to 150 participants. Overall, 30 stories underwent peer review and were published on the institutional website. The focus group participants stated that the reasons why they participated in the CoP were the need to share experience and to tackle new challenges. Conclusion: The CoP was found to be useful for improving professional practice by strengthening professional identity and contributing to the production of new knowledge.

## 1. Introduction

The problem of workplace injuries in Italy is serious. Injuries still occur despite the application of well-defined laws and safety norms [1] and despite the use of enforcement actions by the Workplace Safety and Prevention Services (WSPS) of local health boards [2]. To get to the base of the problem, we need to better understand the proximal as well as the distal causes rooted in the context and social conditions. We also need to grow a culture of safety by increasing the spread of information about little known workplace injury investigations. These purposes were pursued by the US National Institute for Occupational Safety and Health (NIOSH), which implemented the Fatality Assessment and Control Evaluation (FACE) program for the prevention of occupational fatalities in 1982 [3,4]. The FACE Program aims to prevent job-related injuries and deaths by:

- Investigating selected fatalities;
- Identifying hazards;
- Developing workplace prevention recommendations;
- Sharing recommendations with employers, safety professionals, and workers.

The FACE Program identifies prevention strategies based on in-depth investigations of selected types of deaths. Each investigation results in a report that describes the incident and includes recommendations for the prevention of similar fatalities. NIOSH has finalized reports on more than 1900 occupational fatality investigations and has posted them at www.cdc.gov/niosh/face (accessed on 13 August 2021). Detailed incident descriptions and recommendations can be critical for designing injury prevention measures, including safety policies and procedures, engineering controls, and other aspects of the safety climate. Therefore, the investigation process and the investigators’ abilities become vital steps in delivering in-depth information that uncovers root causes, not just direct causes. In fact, the usefulness of recommendations is highly dependent on the insights provided throughout the initial investigation process [3,4].

It is crucial to find the right tools for using the rich documentation of the injury investigations carried out by the OSH inspectors and for promoting a process of knowledge transfer and exchange. The story-based approach and the model of Community of Practice seem to respond to these needs [5,6].

### 1.1. Story-Based Approach

Scientific studies consider storytelling an effective information source when coupled with technical–scientific evidence. This tool can promote the structuring of a relationship between evidence-based knowledge and the field experience of the people to whom it is addressed [7].

Stories help people remember critical elements and, like many children’s stories/fairy tales, stories can become life lessons [8]. In the OSH field, the description of what went wrong and caused an injury promotes a process of collective discussion for finding solutions to avoid similar injuries in the future [9]. The story-based approaches may have behavioral advantages over purely informational communications [5]. Furthermore, storytelling can provide additional information on contributing factors necessary for the identification of injury prevention strategies [10,11].

### 1.2. Community of Practice

The concept of “Community of Practice” (CoP) was developed by Etienne Wenger and Jean Lave in the 1990s [6].

Communities of Practice and situated learning are social groups that have the objective of generating organized, quality knowledge to which each group member has access. The aim is to achieve collective improvement. Persons participating in this type of organization strive for creating a model of sharing in which there is no room for competition. Communities of Practice foster the development of professional identity and sense of belonging [6].

Starting from Wenger, a scientific debate developed on the definition of CoP that highlighted the multiplicity of experiences and theoretical approaches regarding this concept. A line of research, in particular, shifted the focus from the first term, “community”, to the second, “practice”.

“Stressed in the former case is that community constitutes the context and the community pre-exists its activities. In the latter, it is the activities themselves that generate a community in that they form the “glue”, which holds together a configuration of people, artefacts, and social relations. Moreover, this shift also changes the emphasis placed on knowledge: in the former case, learning is viewed as access to and the mastering of expert knowledge possessed and nurtured by the community; in the latter, the attention is directed at the practical knowledge contextually employed during performance of a practice” [12] (p. 523).

The CoP model can provide opportunities for removing professional and organizational barriers. It may also be effective in supporting learning among newly hired staff and in translating and sharing tacit knowledge and know how [13]. As a valuable resource for skills development and a driver of the implementation of evidence-based skills, the CoP model offers novel ways to organize collaboration in response to the challenges of complex systems [14].

To our knowledge, there are no examples in the literature on the use of the CoP concept in the field of occupational safety and health (OSH).

A systematic review that evaluated the efficacy of CoPs for improving clinical practice in health care found notable differences in the composition, objectives, and tools [15]. The feature common to all CoPs is the intention to facilitate learning and to exchange information and knowledge that improves professional practice. Recent studies included in the review have tried to evaluate the impact of CoPs on improvement of performance in health care. The outcomes of a CoP can differ widely, including acquisition of new skills; removal of professional, geographic, and organizational barriers; reduction in professional isolation; and facilitation of the implementation of new processes and technologies.

### 1.3. The Current Study

Based on these considerations, in 2012, we developed a project in which OSH inspectors rewrote injury investigation reports using storytelling and shared the stories on the https://www.storiedinfortunio.dors.it website (accessed on 13 August 2021). The stories followed the narrative structure described elsewhere [16]. Particular attention was given to describing the indications for prevention, i.e., the experiences, procedures, and actions necessary for injury prevention.

In the two-year 2012–2013 period, we held two seminars for OSH inspectors of Piedmont (northwest Italy). The objectives of the seminars were to train inspectors to identify significant workplace injuries, to write effectively, to identify key elements that make a story interesting, and to include information gleaned from witnesses’ statements to flesh out the details on critical points in reconstructing an injury narrative. After the two seminars, each inspector had to write an injury story according to the narrative structure shared starting from his own injury investigation. Each story finished with a paragraph in which the author(s) reported the indications for injury prevention. The results of this first phase of the project have been shown elsewhere [16].

Two years after the project began, OSH inspectors asked the project group to improve their indications for injury prevention through peer review, with the aim of overcoming the personal subjectivity [17].

The model of the Community of Practice (CoP) according to Wenger seemed the most suitable tool to respond to this need.

The aims of this paper are:

- To describe the setting up of a CoP of OSH inspectors in which they could share knowledge and formulate best practices for workplace injury prevention.
- To evaluate the motivation of OSH inspectors to participate in the CoP and the effects of participation on their professional practice.

## 2. Materials and Methods

In 2014, we supported the constitution of a CoP to integrate and approve, by a peer review process, the indications for prevention specific to each individual injury story. In the following paragraphs, we describe the process of elaboration of best practices by CoP members, the evaluation plan for assessing the motivations of OSH inspectors for participating in the CoP, and the effects of participation on their professional practice.

### 2.1. Elaboration of Best Practices for Injury Prevention

Each CoP meeting was structured as a workshop in which two injury stories were submitted to peer review by the CoP participants. The stories were selected according to the criteria of originality and complexity in describing prevention measures. Each story’s author(s) provided the group with support documentation: injury site report, photo documentation, and eyewitness accounts. The injury narratives and related documentation were rendered anonymously and presented without revealing the authors’ identities (blind), and the stories were without the parts in which the authors described the prevention means that could probably have averted the injuries.

**Figure 1:**
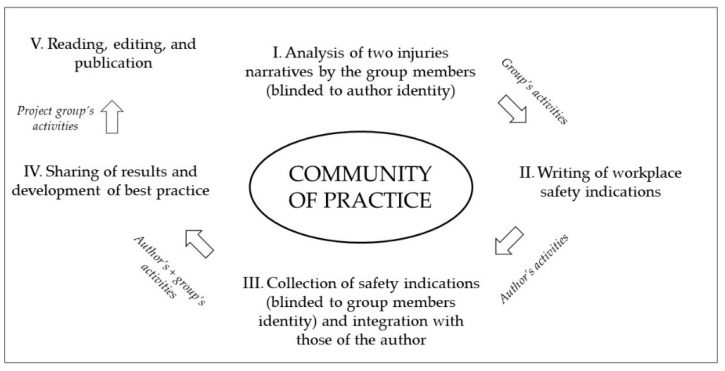
Process of development of the best practices for injury prevention during CoP.

Figure 1 shows the different steps of the development and approval of the best practices. After forming two groups, the participants worked on indications for injury prevention by analyzing the documentation and discussing the case (steps I and II in the figure). Then the story author(s) received the group’s safety indications, unaware of the identity of the group members (double blind), compared them with his/her/their own (step III in the figure), and discussed the feedback with CoP members during the next CoP meeting. After the CoP meeting, the author(s) rewrote the best practices section of his/her/their story, taking into account the results of the discussion, and sent the story to the project group (step IV in the figure). The project group read, edited, and finally published the story on the institutional website (step V in the figure).

### 2.2. Evaluation

We used two instruments for the evaluation of the motivation of OSH inspectors to participate in the CoP and the effects on their professional practice: a focus group [18] and an open-ended questionnaire. All the participants were informed about the aims of the study, and their written informed consents with sociodemographic data were obtained before the study began.

### 2.3. Focus Group

The focus group (FG) consisted of seven OSH inspectors selected from all the participants in the project. Participants in the FG were enrolled on the basis of two criteria:

- Geographical representativeness.
- Inspectors who had participated in all CoP meetings and were able to bring a more in-depth contribution to the discussion, according to the theoretical sampling method used in qualitative research [18].

Participants belonged to five different WSPS and worked as OSH inspectors for at least ten years; the mean age was 45 years, and one participant was a female.

The FG was formed in September 2016 and was conducted by a team of experts not involved in the project. The topics concerned the inspectors’ perception of the utility of their experience in the CoP, critical aspects, and potential developments. The group discussions were audio-recorded and reported in a text. The framework for the FG discussion is shown in Appendix A.

The FG lasted about two hours, was very lively and participatory. The discussion format was not directive and left ample room for interaction among the participants. A topic was considered “saturated” when all operators were interviewed and the new contributions did not add original elements to the discussion [18,19].

The experts who conducted the FG and a qualitative research expert carried out the analysis of the text. They identified concepts consistent with the topics under study and the level of sharing between participants of the FG [18].

The text of the FG was analyzed using an Excel grid, attributing the responses of participants to the themes of the FG (motivations for participating in the project, added value, repercussions on work, critical issues, and possible developments), reporting for each area the acronyms of the inspector who had expressed that assessment. In this way, contributions consistent with the FG’s survey objectives were identified and their level of sharing by FG participants could be measured. The Excel grid is shown in Appendix B.

Text analysis was supported by a text mining activity, with a reduction of the texts to their own lemmas and a calculation of their frequency, through the use of FreeLing software, an open source language analysis tool suite [20,21].

### 2.4. Open-Ended Questionnaire

During a CoP meeting in February 2017, we asked the participants to write the answer to the following question: “In your experience, what are the criticalities and opportunities of using the Community of Practice model for examining workplace injury prevention?”

A total of 29 OSH inspectors who had not participated to FG carried out the task. A qualitative research expert carried out the analysis of the text using the same methodology used for the FG.

## 3. Results

### 3.1. Setting up of a CoP for the Implementation of Best Practices

OSH inspectors who participated in the two seminars on storytelling and wrote the injuries stories were invited to participate in a meeting with the aim of discussing and sharing indications for injury prevention. During the meeting, the CoP model according to Wenger was presented. We agreed with the group of participants the aims of the CoP, the working mode, the frequency of the meetings, and the venues. Twenty participants joined on a voluntary basis. They belonged to different Workplace Safety and Prevention Services of local health boards, and each of them involved other colleagues over time. The CoP meetings were organized in various locations within the region to facilitate the participation of OSH inspectors. Participation in the CoP meetings allowed learners to obtain training credits, which are mandatory for OSH inspectors.

Over the years, we held three other seminars on storytelling to involve and train new OSH inspectors. As a result, the number of OSH inspectors who wrote the stories and the participants in the CoP increased.

Between 2014 and 2021 the CoP met 18 times, about 3 times a year, in different locations throughout the region.

At the beginning of the project, the network comprised OSH inspectors of the regional health boards in Piedmont, but later it expanded to include those of other regions of the country, plus newly hired staff. The inspectors who participated voluntarily in the CoP had directly followed the injury investigations.

On average, each meeting was attended by 30 inspectors. A total of 150 OSH inspectors attended at least one CoP meeting; 15 inspectors attended all of the workshops. Overall, 30 stories submitted to peer review, accompanied by a best practices section, have been published on the institutional website and are downloadable free of charge. Since 2013, the webpage has had more than 10,000 visitors, with more than 65,000 pages visited.

### 3.2. Evaluation through Focus Group

#### 3.2.1. Motivation for Participating in the Community of Practice

Most OSH inspectors in the focus group stated that the main reasons for participating in the CoP were the need to share and compare their experiences of carrying out investigations in workplaces where an injury occurred, the need to take on new challenges by rewriting the report in narrative form, and the need to share the doubts inherent in the investigation.

As one participant stated *“This peer community allows us, in a place other than our office, to get involved. It also help us to see events even with the eyes of the other and we realize that there is not only one way of acting, there are also others.”*

The group underlined that this type of discussion was necessary, as working situations have become more complex, not so much in the way that they exist, but rather because of the changes in the organization of work owing to the increase in subcontracting and outsourcing of services and labor. As one participant stated, *“The other question, in my opinion, regards how our working world has evolved. Thirty years ago, you could carry out an investigation because the worker was there, had been working at the same job for 20 years, and got his finger crushed because there was no safety guard on the press. You could write up your report, have the situation fixed, and that was the end of it. It was simple. Now when you come into a company, the worker works for a cooperative that belongs to another company that works in another area.”*

#### 3.2.2. Effects on Professional Activities

Regarding the effect on one’s own way of working, most of participants in the focus group discussed the changes in the method of injury analysis, with greater attention to situational and relational aspects of the workplace. A participant stated, *“Now, having attended these meetings, I’ll approach the injury investigation by looking at it from different perspectives, not just from one, but rather trying to understand what happened and what was behind it.”*

**Figure 2:**
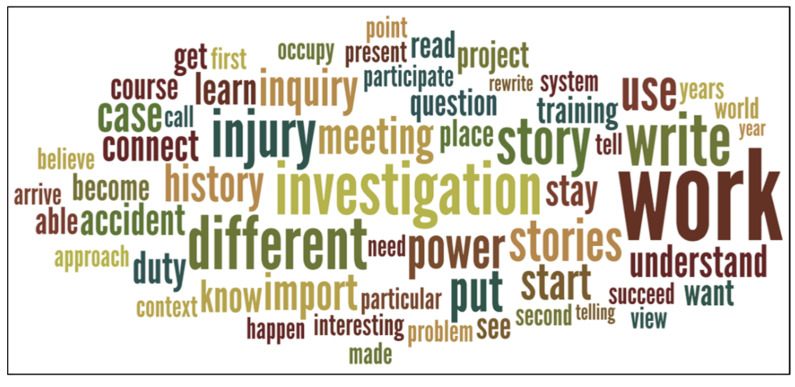
Word cloud of 60 most frequent headwords in the areas covered by the focus group: motivation, level of criticality, development.

Figure 2 presents several recurrent topics that show how the CoP contributed to revealing different ways of conducting injury investigation and that the CoP was also a tool for learning and knowing.

Among the most used terms in the focus group, next to those that were part of the daily lexicon of the inspectors, such as “work, investigation, injury”, other concepts related to process aspects and impact on professional skills are relevant.

The recurrence of terms such as “meeting, connect, power, system” highlights the importance of the CoP in overcoming the isolation in which inspectors normally work. Bringing together specialists and building relationships and professional networks has an impact on the awareness of one’s own “power” within the “system”.

Words such as “learn, know, able, understand” underline aspects related to the acquisition of new “skills” and “knowledge” that encourage a new “approach” to their professional activity of “investigation”.

In the transition from indications for injury prevention written by the author to the best practices shared by the CoP, we found a significant enrichment in the way of approaching the topic.

To give an example, we report in Appendix C the injury story “One job, two masters”, involving an employee of a cooperative under contract who cut her thumb while working on a pastry production line. After the discussion and peer review by the CoP, in the best practices section, two elements were added to the original indications for prevention written by the author(s) of the story.

The first concerned the assessment of the risks due to the lack of cooperation between the contracting company and the cooperative under contract, other than the unclear division of labor between the two firms.

The second concerned the need to supplement the workers’ training with a more detailed exchange of information on the operating modes between the contracting company and the workers of the cooperative.

Below we report an excerpt taken from the best practices section of the story (see Appendix C):

*“Collaboration between employers in writing up the Interference Risk Assessment Document, i.e., an assessment statement of interference risk, is a formal normative requirement and an important aid in the management of safety as regards external contractors. The document lists the risks associated with different activities performed within a given workplace area, the minimal precautions, and the rules for eliminating or minimizing the consequences of such risks (…). The cooperative did not work autonomously. The skilled workers of the cooperative and of the bakery worked together. This situation contrasted with the supplier’s service contract stipulated by both parties.”* [...]

*“In the present case, exchanging information on how to work on the production line, the risks involved, what to do in case of problems, and clearly establishing the roles and duties of workers could have made the injury more difficult and less likely to occur.”*

Another benefit of the CoP process was the strengthening of professional identity and increased recognition by interlocutors not belonging to the same area. A focus group participant stated, *“I think that underlying our interest in participation is, somehow, the desire for redemption. We’re getting on in years, with the burden of experience weighing us down, and after so many you just want to unload and become a new person. It’s a way to get free, to rejuvenate, as an approach, as an emotion.”*

An unexpected result of this process concerns the greater awareness of the inspectors with respect to their professional role: the need to become a part of the “stories” themselves through the sharing of their experiences, difficulties, and emotions.

*“In writing the stories, personally, we can put all those aspects, opinions, sensations only felt, perceived, that in injury investigations we never put.”*

This “empowerment” process contributed to the rise of a new awareness of their professional identity—from “culprit hunters” to “prevention agents”, able to increase the safety culture.

*“Now, after these meetings, I try to approach the injury investigation from different points of view, not following a single reasoning aimed to find the culprit, but trying to understand the context where the injury happened and how it could have been avoided.”*

#### 3.2.3. Critical Issues

The majority felt that the main criticalities were the uneven attendance by participants at the meetings. In the beginning, the focus was on judging colleagues’ work as if in a contest, but then later this problem was overcome, and a constructive dialogue could begin. Another criticality was the time constraint: writing up the injury stories and attending the meetings took up time and resources that management often does not recognize. Lastly, the discussion centered again around violation of norms; the aspects of the context, albeit thought to be important, were rarely considered.

#### 3.2.4. Potential Development

The focus group underlined the need to increase awareness of risk not only among workers but also in the general public. One participant suggested transforming the stories into comic strip format, while another participant felt that the injury story narrator should be the worker who was involved in the injury, in a kind of flashback narrative of how the facts were reconstructed, the difficulties encountered, and how they were resolved.

Another comment was that “*We are never there when it happens. We tell stories but are never present. We are storytellers.*”

With regard to broadening experience to include others who deal with workplace safety, the majority of the inspectors felt that it was still too early since it would make the sharing process among peers difficult due to the different roles they have.

### 3.3. Evaluation by Open-Ended Questionnaire

With regard to the questionnaire results, most of the inspectors stated that they found no criticalities; four mentioned the time constraints, and four others said that legitimation by management was missing. As concerns opportunities, most of the questionnaire responses (20/54) were consistent with the FG statements: the CoP model offers ample opportunities for exchange of views with colleagues for generating knowledge and approving indications for prevention.

Table 1 presents the questionnaire responses classified according to conceptual categories.

## 4. Discussion

The CoP model seems to respond to the need of OSH inspectors to improve the indications for injury prevention. It consents to share collective knowledge and to design safety prevention measures that, through peer-reviewed approval, become best practices transferable to other safety prevention specialists. These best practices constitute the final product of the CoP, obtained through reification, as described by Wenger, who identified the “material entity” as the essential outcome of a CoP [6].

The sharing of the practice of narration of injuries and the discussion on preventive means that occurred in our project fostered a learning process, individual and collective, that motivated the participation of OSH inspectors to the CoP and constituted “the glue” [12]. The CoP described in this study provides its members with a useful tool for self-learning and the acquisition of new knowledge that comes not from reading norms and manuals but rather from taking part in discussion and sharing experiences. In particular, the use of the double blind method during the different steps of the CoP helped to vanquish the prejudice, often implicit, from mutual competence and experience.

The experience of the CoP, in a highly individualized and fragmented work context, produced a strengthening of their professional role, favoring a growth in the level of “trust” towards their colleagues and the working group. The increase in participants from 20 to 150 during the course of our project was a sign of success in building a network of professionals. Indeed, the organization of CoP meetings in different locations within the region, together with the training credits, influenced this result by encouraging the participation of OSH inspectors who were more reluctant. In addition, the core of 15 OSH inspectors who participated in all 18 meetings joined the project group, thus encouraging the involvement of their peers. The inclusion of newly hired staff fostered the learning process and the transfer and sharing of knowledge among CoP participants with different levels of work experience and professional profiles.

The CoP was found to be useful for the improvement of the professional practice of OSH inspectors. Through the analysis of solutions and problems, the CoP members were able to study the causes of workplace injuries in relation to the context and the organization, factors usually ignored because they are not directly connected to the violation of norms. As in the FACE program, they overcame an approach based on fault or blame. This evolution provided a better understanding of accident causation, allowing the CoP members to pursue best practices that went beyond repetitive and general instructions [3,4].

Since this CoP has voluntary membership, which till now involved only a part of the OSH inspectors, the problem arises with regard to the recognition and legitimation of this activity by the WSPSs in which the CoP members are working. If the CoP cannot be “imposed” by the management, it can be recognized as a “formative” process, useful for improving the quality of work of OSH inspectors, also with regard to organization and motivation aspects. In this sense, the organization of a CoP meeting three times a year with no more than 30 participants was a sustainable solution.

The elements that emerged from our focus group and open-ended questionnaire appear to corroborate the coherence of this model with the theoretical underpinnings of the CoP concept with regard to the results (production of best practices), process (bottom-up approach), and redefinition of the sense and professional identity [6].

To our knowledge, this is the first study on the use of CoPs in the field of OSH, so it is difficult to compare this study with others. Although CoPs have been used in health care since the mid-1990s, few efficacy evaluation studies have been conducted due to the difficulty in defining what exactly constitutes a CoP. Since the concept of the CoP is evolving, describing its characteristic features has been elusive. Analysis of the available literature has pinpointed several key aspects, such as support of formal and informal interaction between newly hired and experienced staff, attention to learning and exchange of knowledge, and investment in the sense of belonging among group members. That said, it is nearly impossible to isolate the effects of a CoP and to determine the relationship of causality between a specific program and the expected results [13,14]. Even if the CoP model is not mentioned among different methods of training evaluated in two recent systematic reviews about OSH training, the relevance of high engagement and interaction joined with noncompulsory participation is coherent with their results. In addition, the authors of the reviews encourage continuation of the training over time [22,23].

A limitation of this study is that the evaluation process was based on qualitative methodologies, which makes the outcomes difficult to generalize. Additionally, the focus group and open-ended questionnaire involved a small number of participants. During the final months of 2016, a similar project with the same approach and training method was carried out in the neighboring region of Lombardy. From the first semester of 2018, new initiatives began to try out the CoP concept with other professionals who deal with workplace safety, including worker safety representatives and heads of prevention and protection services belonging to public and private companies. These new scenarios will make further evaluations necessary that take into account other contexts and different organizations.

Among the future developments, there is the evaluation of the method through an analysis of changes in inspector performance concerning, for example, the quality of workplace injury investigation and reports to judicial authorities or adherence to checklists for supervisory activity using pre–post evaluation models to understand the value of a CoP within an organization. The CoP model, in this case, could be counted as a new training method also intended for workers who, studying the documentation and discussing the injury case or a specific risk situation, work together on preventive solutions. The best practices could be greatly enhanced through CoP meetings involving members with different roles in the OSH field, such as inspectors, worker representatives, and heads of prevention and protection belonging to public and private companies. This seems to be one important way to move research into practice.

## 5. Conclusions

The CoP was found to be useful for improving professional practice by strengthening professional identity and role and contributing to the production of new, shared knowledge. The process for the development and approval of best practices described in this study seems to be a promising method that needs to be deepened in future OSH research. The adherence to the CoP was not imposed by management, the cooperative learning and the realization of a tangible product, such as the best practices, were the key elements that motivated OSH inspectors to participate. The CoP can be suggested as a method of cooperative learning both for operators who are already working and especially for those who are new hires.

We believe that the project outcomes can be transferred, integrated, and further developed in other areas and settings.

## Figures and Tables

**Table 1:** Questionnaire responses classified according to conceptual categories.

Opportunities	Number of Responses
Exchange of views with coworkers for producing knowledge and approving indications for injury prevention	20
Circulate information to others: students, OHS specialists, other stakeholders	8
Published stories: resource for persons working on occupational health—employers, heads of injury prevention and protection services, consultants, worker safety representatives	4
Occasion for professional growth: different way to examine an injury event	4
Knowledge of workers’ experience	4
Means to express the emotions of all involved	3
Sharing/approval of best practices	3
Gain knowledge of different types of approaches	2
Use of published stories following discussion among coworkers to standardize injury investigation	2
Gain knowledge of technical situations for use in similar cases	2
Creation of an archive of stories/best practices approved by a CoP	2
Total	54

## Appendix A

Draft discussion framework for the focus group

1. Perception/evaluation of participation in the community of practice (CoP) project.
  All of you have been writing and dispensing injury narratives for several years:
  - What were your reasons for participating in a CoP?
  - How did participating in CoP benefit/enhance your experience of writing injury narratives?
  - Has your experience in the CoP met your expectations?
  - What are the main criticalities and limitations?
2. Concerning the effect on your professional activities and in the service.
  What are the main outcomes for your professional practice and for your services?
  - With regard to process: sharing, comparison with colleagues, development of sense of belonging and professional identity.
  - With regard to outcomes: enrichment of your professional knowledge/skills (e.g., greater attention to the context, way in which the injury occurred, organizational elements, profile of the individuals involved).
  Have you had the occasion to use/evaluate this experience in your professional practice (site inspection, investigation, and training)?
3. Potential developments
  - What can be improved?
  - Would it be interesting/useful to engage your colleagues in this experience?
  - Would it be interesting/useful to engage other professionals involved in company safety?

## Appendix B

GRID USED FOR THE FOCUS GROUP

Participants to the focus group

1. OSH inspector
2. OSH inspector
3. OSH inspector
4. OSH inspector
5. OSH inspector
6. OSH inspector
7. OSH inspector

REASON TO PARTICIPATE IN COP		
Keywords	OSH inspector	text
	1	
	…	
EFFECTS ON YOUR PROFESSIONAL ACTIVITIES		
Keywords	OSH inspector	text
	3	
	…	
ENRICHMENT OF YOUR PROFESSIONAL KNOWLEDGE		
Keywords	OSH inspector	text
	2	
	…	
CRITICAL ISSUES, LIMITS, DIFFICULTY		
Keywords	OSH inspector	text
	7	
	…	
POTENTIAL DEVELOPMENTS		
Keywords	OSH inspector	text
	5	
	…	

## Appendix C

ONE JOB, TWO MASTERS

What happened?

An employee of a cooperative cut her thumb while working on a pastry production line.

Who was involved?

Bianca, a 42-year-old worker, Macedonian national, mother of two boys attending mandatory school. She lives with her family in an apartment in a village in the Langhe hills near the city of Asti (Italy). Bianca joined the services cooperative in 2007 under a seasonal contract as a packing machine worker.

*“I’ve been employed with Drago [the cooperative] for about 5 years.”*

In 2008 her contract was changed from a temporary to a permanent one. Although she has lived for several years in Italy, Bianca understands and speaks little Italian.

*“I don’t understand and speak very good Italian and I don’t read.”*

Where and when

The injury occurred in the province of Cuneo, one afternoon in December 2010, in the production department of an industrial bakery. A subcontractor agreement existed between the bakery and the cooperative for the services of manual packaging of bakery products, wrapping, labelling, palletting, and porterage. The work is divided in three shifts, with two daytime shifts during which 15 cooperative employees, on average, and about 10 bakery employees are present, and one night-time shift. Bianca had been working the night shift for 3 years.

*“For the past three years I’ve worked steady for 6 months at the Le Dolcezze srl company in Forno. For the other 6 months of the year, I’ve worked in other factories that the cooperative has contracts with. Le Dolcezze srl makes biscuits and puff pastries. The cooperative employees work in various different departments of the bakery on various jobs: packaging, boxing, labelling, stock movement in the warehouse, on the production lines, and cleaning. All these jobs are carried out by the employees at Forno. Work is done in three shifts and I always work the night shift.”*

What was being done?

During the weekend, Bianca was told by the wife of the president of Drago [the cooperative] that the following Monday she would work the afternoon shift at the bakery. When Bianca arrived at the bakery, Valeria, the cooperative foreman, sent her to work at one of the three puff pastry production lines.

The production lines stand next to each other. They differ in output capacity, form of the product, and year of construction. Each line is fed by bobbins of dough, i.e., a long sheet of dough wound around the bobbin. As the bobbin unwinds, the dough is stretched out on the band, folded over on itself, then cut into small pieces to give the product its final shape. On exiting the tunnel oven, the products are packaged. All these operations are performed by the fully automated line. Bianca’s job was to make sure everything proceeded smoothly up to the entrance of the tunnel oven.

*“Saturday or Sunday, I don’t remember which, Ms. Antonella called to say I had to do the afternoon shift at Forno. When I came to the bakery on Monday, my colleague Valeria, an employee with the cooperative, a foreman, told me to go work on the line that cuts the dough and sends it into the oven. The bakery has three lines: the first we call the ‘big’ line, the second the “little” line that stands between the other two, and the third the ‘new’ line. My job was to make sure the line worked properly and to solve problems if there were any.”*

At a certain point

Shortly after the afternoon shift had begun, Bianca noticed that clumps were forming around the area where the dough is cut and ruining the cut and the form of the dough. She tried to call the maintenance man but could not find him. So she decided to try to fix it herself. She took a spatula and, without stopping the line, began to scrape away the dough clumps that had collected. The cutting knives descended and her right thumb came into contact with the knife blade.

*“After about a half an hour at the line on Monday, I noticed that the dough was clumping near the knives and that they weren’t cutting the way they should. I tried to call Mr. Massimo, the bakery mechanic who takes care of these problems, but no one answered. So I took a spatula and tried to scrape away the clumps of dough that were forming. But while I was doing that my right hand got into the cutting area and the knife cut off the end of my thumb.”*

Bianca was taken to the emergency department for treatment.

*“I looked for paper towels to close the wound, since it was bleeding badly, and I ran over to Valeria to show her what happened and she called the office so they could call the emergency services to take me to the emergency department. The people in the office said to wait because they had called Mr. Giacomo and wanted to wait till he came. So my colleague called another cooperative employee, Zako, and told him to take me to the hospital. We went in my car and Zako accompanied me to the emergency department. There they diagnosed amputation of the distal phalanx of the right thumb, medicated the wound, and placed some stitches.”*

What came out of the investigation?

Comparison between the situation in the bakery at the time the injury occurred and Bianca’s initial account differed as to whether there were safety guards around the cutting area or not. Bianca stated that there were no safety guards, whereas later inspection of the injury site revealed there were. In order to precisely determine the injury dynamics and the point at which it occurred, several inspections had to be carried out at the bakery and then integrate Bianca’s first statement with her later one. Furthermore, because of Bianca’s difficulty in understanding Italian, her statements were often confusing and contradictory.

*“At the bakery there are three production lines: the first one we call ‘big’, the second we call ‘small’, and in between is the third we call ‘new’. I had to work on the ‘new’ line.”* Witness statement.

*“In addition to the statements I have already made, I wish to point out that I injured myself on the ‘small’ and not on the ‘new’ line, as I had erroneously stated.”* Witness statement.

“[…] *I tried to call Mr. Massimo, who is the mechanic of the Le Dolcezze srl bakery and who deals with these problems, but no one answered.”* Witness statement.

*“I wish to point out in addition that Mr. Massimo, who I mentioned in previous statements, is not the mechanic of the bakery but rather deals with production.”* Witness statement.

Despite the misunderstandings and contradictions (presence of safety guards on the production line before or after the injury), the safety guards on the ‘small’ production line, site of the injury, were insufficient. The cutting area was enclosed on its sides by a protection grille but the exit area was closed by a tilting metal frame door with a transparent Plexiglas window. The door had no safety sensors and the closure was secured with a thumbscrew. This mechanism can be easily mishandled and needs no key or other tool to open it. There were, however, emergency buttons in the cutting area which, had they been activated, could have stopped the production line. The other two production lines were sufficiently equipped with safety systems: the cutting area was enclosed on all sides by a metallic protection grille and interlocking devices associated with guards. The line Bianca was injured on was the only one with a Plexiglas guard.

The Le Dolcezze srl company agreed to lend the Drago cooperative the ‘small’ production line for temporary use. This line had safety issues, however. In addition, the bakery and the cooperative employees would alternate using the line. The weekly work shifts were planned by the bakery’s head of production, specifying the number of cooperative employees that were to work during which shift on which production line. The president of the cooperative decided which workers would be sent to work at the bakery.

How it ended

The National Institute for Insurance against Accidents at Work (INAIL) awarded Bianca disability status of 3%. For many months after the injury, she was not called by the cooperative unless for occasional work. The employer of the cooperative was accused of having exposed workers to equipment not compliant with safety regulations and to have miscalculated the risk of injury. Both claims were settled. The managing director of Le Dolcezze srl was accused of having lent an unsafe piece of equipment. The company revoked the loan agreement but did not pay the sanction. The formal and ‘de facto’ employer of the company was accused of having exposed his employees and those of the cooperative to equipment not conform with safety requirements and not to have correctly evaluated the risks involved. The prescriptions were not fulfilled.

Best practices

These best practices were developed by a community of practice from a collection of injury narratives that were then revised by the story author.

Maintaining equipment and machinery safe for use according to current norms of workplace health and safety is essential to minimize the risk of injuries. This should be coupled with a method that best identifies and evaluates the risks present in the workplace so as to select the most appropriate prevention and protection measures. Furthermore, people need to be trained and informed about workplace risks and correct work practices.

Particular attention should be taken in cases involving foreign workers who have a poor or no knowledge of Italian. In such instances it is difficult to determine what type and degree of training they may have received. In the present case, poor comprehension led to misunderstandings and contradictory statements. It would therefore be preferable to use the simplest language possible, avoiding technical, legal, and regulatory terminology.

Another important aspect of this case is the presence of subcontractors working within a company. Collaboration between employers in writing up the Interference Risk Assessment Document, i.e., an assessment statement of interference risk, is a formal normative requirement and an important aid in the management of safety as regards external contractors. The document lists the risks associated with different activities performed within a given workplace area, the minimal precautions, and the rules for eliminating or minimizing the consequences of such risks. Again, in the present case, exchanging information on how to work on the production line, the risks involved, what to do in case of problems, and clearly establishing the roles and duties of workers could have made the injury more difficult and less likely to occur.

The use of worker cooperatives is very often a cover for furnishing manual workers under a seemingly regular service contract. This arrangement is highly convenient for businesses as it ensures workforce flexibility, wherein the number of cooperative workers sent to a company matches the momentary demand for labour. Another convenience for the company is that the responsibility for worker safety falls upon the cooperative employer. In the present case, the Le Dolcezze srl company and the Drago cooperative had a contract for the rental of a specific area of the production department, where the cooperative would have its workers work. In addition, there was also a temporary loan agreement for use of some of the bakery equipment, including the three production lines. Actually, however, the cooperative and the bakery workers worked together in various parts of the production department of the bakery. In addition, the three production lines were used by all workers, indiscriminately of work shift, equipment, and specific job assignments. The cooperative did not work autonomously. The skilled workers of the cooperative and of the bakery worked together. This situation contrasted with the supplier’s service contract stipulated by both parties. In such situations, clarification on types of employment contracts needs to be sought from the staff of the provincial Department of Labour.
